# Extracellular Vesicles in Alzheimer’s Disease: Mechanisms, Biomarkers, and Therapeutic Engineering

**DOI:** 10.3390/ijms27093974

**Published:** 2026-04-29

**Authors:** Lian Wang, Liwei Mao, Xuemei Zong

**Affiliations:** 1 Department of Neurology, Institute for Cerebrovascular and Neuroregeneration Research (ICNR), Louisiana State University Health Sciences Center, 1501 Kings Highway, Shreveport, LA 71103, USA; wanglian9369@hotmail.com (L.W.); 15951911332@163.com (L.M.); 2 Department of Neurology, Medical College of Georgia, Augusta University, 1120 15th Street, Augusta, GA 30912, USA

**Keywords:** extracellular vesicles, Mesenchymal stem cells, Noncoding RNA, Alzheimer’s disease, Bioengineering

## Abstract

Extracellular vesicles (EVs) are nanoscale membrane-bound particles that mediate intercellular communication by transferring proteins, nucleic acids, lipids, and metabolites. Increasing evidence implicates EVs in Alzheimer’s disease (AD) pathogenesis through the propagation of amyloid-β, tau, and neuroinflammatory signals across neural and glial networks. In parallel, EVs isolated from biofluids have emerged as promising sources of disease-associated biomarkers and potential therapeutic carriers. This review aims to synthesize current evidence on EV-mediated mechanisms in AD, evaluate the diagnostic value of EV-associated biomarkers, and discuss emerging EV-based and bioengineered therapeutic strategies. We summarize how EVs derived from neurons, astrocytes, microglia, and peripheral cells contribute to amyloid-β and tau spread, neuroinflammation, synaptic dysfunction, and metabolic stress in AD. Disease-associated alterations in EV cargo from blood, cerebrospinal fluid, and urine are critically assessed for biomarker applications. We further highlight advances in EV bioengineering, including cargo loading, surface modification, targeting strategies, and modulation of EV biogenesis. Finally, key translational challenges—such as EV heterogeneity, biodistribution, immune clearance, and standardization—are discussed to define future directions for leveraging EVs as diagnostic and therapeutic platforms in AD.

## 1. Introduction

**Figure 1:**
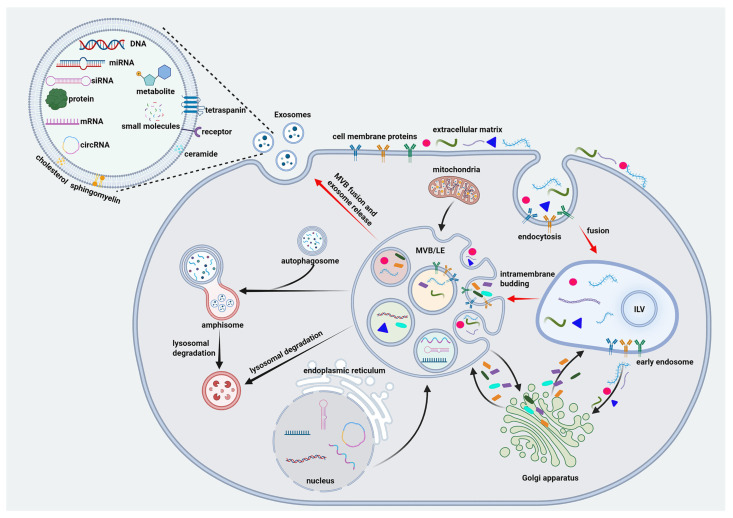
Exosome biogenesis and cargo composition. Exosome biogenesis begins with endocytosis, forming early endosomes (EEs) that mature into multivesicular bodies (MVBs) through intraluminal vesicle (ILV) budding. MVBs interact with various organelles to promote cargo sorting and loading. MVBs either fuse with lysosomes for degradation or with the plasma membrane to release exosomes into the extracellular space. Exosomes are enclosed by a phospholipid bilayer enriched with sphingolipids, ceramides, cholesterol, and glycosphingolipids, which contribute to exosome membrane stability, cargo sorting, and cellular interactions. The exosomal membrane also contains tetraspanins, integrins, transport proteins, and immunomodulatory molecules, facilitating cellular recognition and uptake. Internally, exosomes carry cytoplasmic and nuclear proteins, nucleic acids, and metabolites involved in cellular signaling, metabolic regulation, and microenvironmental modulation. Red arrows indicate the process of exosome biogenesis, whereas black arrows represent intracellular interactions.

**Figure 2:**
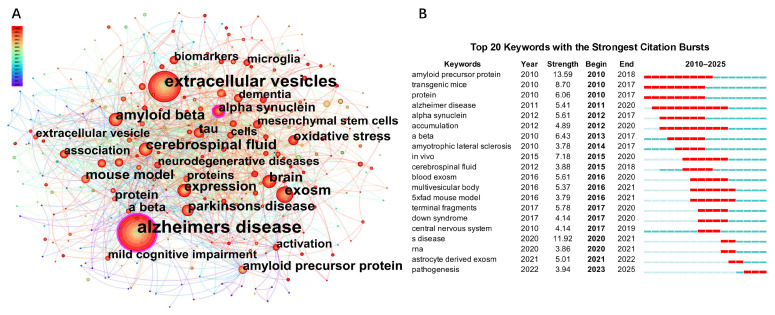
Bibliometric mapping of research trends in EV studies related to AD: (**A**) Keyword co-occurrence network of exosome-related AD research from 2010–2025. Node size reflects keyword frequency, and color gradient indicates publication year (red = recent; blue = earlier years). (**B**) Top 20 keywords exhibiting the strongest citation bursts, illustrating emerging hotspots and temporal trends. Peaks in citation activity (red bars) emphasize shifting research focus from 2010–2025.

Extracellular vesicles (EVs) comprise a heterogeneous population of membrane-enclosed particles, including small EVs, medium/large EVs, and apoptotic bodies, which differ in their biogenesis pathways, molecular cargo, and biodistribution. Accumulating evidence indicates that multiple EV subtypes, rather than a single “exosome” population, contribute to Alzheimer’s disease (AD)-related pathology and biomarker signals. Accordingly, future EV biomarker studies should stratify vesicles by size, density, and marker profiles (e.g., tetraspanins such as CD9/CD63, annexins, and apoptotic markers), rather than pooling all EVs into a single fraction. In the strict mechanistic sense, exosomes refer to EVs generated by the fusion of multivesicular bodies (MVBs) with the plasma membrane, whereas other EV populations, such as microvesicles and apoptotic bodies, arise from direct plasma membrane budding [1,2,3,4]. In the endosome-derived exosome biogenesis pathway, vesicle formation involves endocytosis, generation of intraluminal vesicles, and Rab27A/B-mediated fusion of MVBs with the plasma membrane [5,6,7,8,9,10,11], while some MVBs undergo lysosomal degradation [12,13]. EVs preserve the lipid bilayer topology of parent cells and carry diverse cargos [14], including proteins, nucleic acids, metabolites, and membrane molecules, such as tetraspanins (CD9, CD63, CD81) [15,16], integrins, and MHC complexes [1,17,18], which mediate stability, recognition, and signaling (Figure 1). Uptake of EVs by recipient cells occurs mainly through receptor–ligand interactions [19,20], endocytosis [16,21], membrane fusion [22,23,24], or lipid raft-mediated entry [25,26]. Increasingly, nanotechnology (e.g., magnetic nanoparticles) has been employed to engineer exosome-enriched small EV preparations for targeted delivery [27,28,29]. Nevertheless, the relative contribution of uptake pathways and the translational challenges, such as targeting specificity, immune clearance, and dosing safety, remain incompletely understood and warrant further mechanistic studies. Clarifying these disease-specific biogenesis and uptake pathways and identifying actionable molecular targets will be critical for the rational engineering of EVs as diagnostic and therapeutic platforms in AD. To contextualize recent progress in EV research and its relevance to AD, we performed a bibliometric network analysis to visualize global publication trends, co-occurring research themes, and emerging hotspots (Figure 2).

## 2. Characteristic Changes in EVs in AD

Different types of EVs isolated from various sources display characteristic changes in Alzheimer’s disease, indicating disease-specific pathophysiological events occurring at the periphery and centrally. The contents of these vesicles include proteins, nucleic acids, and lipids, which have been suggested as potential biomarkers and may elucidate the mechanisms associated with AD progression (Table 1). Importantly, these biomarker candidates span different stages of development, ranging from exploratory findings to markers approaching clinical validation.

### 2.1. Peripheral Blood EVs

Peripheral blood-derived EVs offer a minimally invasive source for biomarker discovery, despite their heterogeneous cellular origins, which include contributions from immune cells, platelets, endothelial cells, and potentially brain-derived EVs crossing the blood–brain barrier. Compared with EVs directly obtained from cerebrospinal fluid (CSF), peripheral EVs are more accessible but may provide a less specific reflection of central nervous system (CNS) pathology. Importantly, peripheral EVs are thought to reflect the interplay between systemic responses and central neurodegenerative processes, capturing signals related to peripheral inflammation, vascular dysfunction, and potential brain-to-blood communication in AD. An immunomagnetic EV PCR platform revealed that Aβ42 outperformed p-tau as a diagnostic biomarker, achieving 95% sensitivity and accuracy [30]. The infusion of EVs derived from blood into the hippocampus of AD mice illustrated that these EVs had the capacity for diffusion throughout the brain and localization near the Aβ plaques [57], where they were selectively internalized by microglial cells. Proteomic analysis of plasma EVs indicated some candidate proteins, such as α-1-antichymotrypsin (AACT), complement C4b-binding protein, complement component 9, and immunoglobulin-like proteins, which were found to provide better diagnosis [31,32]. Nevertheless, the heterogeneity of the cells from which these vesicles originate, in addition to differences in their isolation techniques and systemic effects, might account for discrepancies between studies, making it challenging to establish comparability.

### 2.2. Peripheral Blood Neuron-Enriched EVs

Neuron-enriched extracellular vesicles (nEVs), operationally defined as immunocaptured EV fractions enriched for neuronal markers, represent a refined subpopulation of peripheral blood EVs that aims to improve CNS specificity compared with bulk plasma EVs. These vesicles are thought to partially reflect neuronal-derived signals and may provide a peripheral window into neuronal injury, synaptic dysfunction, and protein aggregation processes in AD. However, their true CNS origin remains unproven, as peripheral contamination and the imperfect specificity of neuronal surface markers may contribute to the captured EV population. Levels of Aβ42, total tau, and p-tau are significantly elevated in nEVs from patients with AD and amnestic mild cognitive impairment compared with controls, closely correlating with CSF concentrations and cognitive performance [36,37]. Importantly, these pathogenic proteins measured in nEVs can predict AD progression up to a decade prior to clinical onset and have also been validated in Down syndrome cohorts [38,58], supporting their potential utility as peripheral biomarkers for dementia risk in this population.

Beyond pathogenic proteins, synaptic proteins, such as synaptophysin, synaptopodin, synaptotagmin-2, SNAP-25, and neurogranin, are markedly reduced in nEVs from patients with AD and frontotemporal dementia [39,40]. These alterations are associated with synaptic dysfunction and have been proposed as predictive markers, identifying individuals at risk for AD up to 5–7 years before cognitive decline [41]. Proteomic analyses across healthy controls, MCI, and AD patients revealed progressive, stage-dependent changes in two distinct protein clusters, with complement component 7 being consistently upregulated and the cytoskeletal regulatory protein zyxin being downregulated with disease progression [42]. Furthermore, nEVs from AD patients exhibited increased accumulation of TAR DNA-binding protein 43 (TDP-43), consistent with its involvement in amnestic dementia syndromes, although no significant associations were observed between EV-associated TDP-43 levels and cognitive performance, neuropsychiatric symptoms, or ApoE4 genotype [43].

Notably, many EV-associated proteins and miRNAs detected in blood overlap across AD, Parkinson’s disease, and vascular cognitive impairment, limiting disease specificity when EV biomarkers are used in isolation. This association probably results from shared neurodegeneration and inflammation pathways rather than experimental noise. Moreover, differences in immunocapture techniques, markers, and methods used for the isolation of EVs might be another source of heterogeneity among studies. Overall, all these results indicate that nEVs could be an intermediary between the possibility of obtaining samples from peripheral blood and the disease-related pathogenesis within the brain, but this issue requires additional investigation.

### 2.3. Urine EVs

EVs derived from urine constitute a distal but easily obtainable biomarker source among other types of EVs, indicating molecular profiles that could be generated by either central or peripheral tissues. Even considering the blood–brain barrier, disease-linked EV contents are released into the bloodstream and eventually end up in the urine, offering a circumventive and non-invasive means to study AD-linked events. Therefore, it is possible that urinary EVs can provide indirect information about AD-linked molecular changes rather than the CNS pathology itself. The present data is largely derived from animal studies. In the 5XFAD mouse model, urinary EVs exhibited early molecular alterations, including upregulated acyloxyacyl hydrolase and downregulated neuraminidase-1, annexin 2, clusterin, and lymphocyte antigen 86 [50], preceding amyloid-β plaque deposition. Pathway enrichment analysis showed changes in lipid metabolism and amyloid-β clearance pathways [59]. Despite having certain benefits in terms of stability and noninvasiveness, their location in the peripheral compartment and lack of available human information limit the specific applicability of EVs isolated from urine. In addition, different origins, physiological fate, and methods of detection may contribute to variations in results, thus stressing the importance of standardization and verification in a clinical population.

### 2.4. CSF-Derived EVs

Isolation of EVs from CSF offers an opportunity for the closest biofluid associated with the CNS, and hence is regarded as offering the most disease-specific EVs for changes related to AD. As opposed to other systemic EVs whose signal could be a result of both peripheral and central factors, isolation of CSF-derived EVs captures brain-derived molecular changes. Unlike bulk soluble CSF biomarkers, CSF-derived EVs preserve cell-of-origin information (e.g., neuronal, astrocytic, and microglial), enabling dissection of pathway- and cell type-specific pathology that is not accessible with conventional CSF analytes. Accordingly, CSF EVs are enriched in neuronal and glial cargos, including Aβ, p-tau species, synaptic proteins, and inflammatory mediators, and show stronger correlations with brain pathology than blood-derived EVs. However, CSF collection requires lumbar puncture, which limits scalability for population-level screening despite its high diagnostic precision.

In addition, while CSF-derived EVs offer greater CNS specificity, differences in isolation protocols and analytical approaches may still contribute to variability across studies, and their clinical utility is likely to be complementary to, rather than replacing, peripheral EV-based biomarkers.

### 2.5. Glia-Derived EVs

Glia-derived EVs, including those released by astrocytes and microglia, represent a functionally distinct subset of EVs that can be detected across multiple biological fluids (e.g., CSF and peripheral blood) and play central roles in neuroinflammation and the intercellular propagation of neurodegenerative pathology. Unlike EV classifications based solely on sample origin, glia-derived EVs are defined by their cellular source and functional contributions to disease processes, particularly in modulating immune responses and the propagation of pathological proteins in AD. Astrocytes and microglia actively release EVs, including endosome-derived exosomes, that play central roles in neuroinflammation and the intercellular propagation of neurodegenerative pathology. Astrocyte-derived exosomes from patients with AD exhibit elevated complement effectors (C1q, C3b, C5b–9) and reduced regulatory proteins (CD59, CR1), indicating complement hyperactivation [52]. This imbalance becomes more pronounced in advanced AD, suggesting progressive dysregulation of astrocyte-mediated immune signaling. In parallel, astrocyte-derived exosomes carry increased levels of β-site amyloid precursor protein-cleaving enzyme 1 (BACE1) and soluble amyloid precursor protein β (sAPPβ), implicating astrocytic exosomal pathways in amyloid-β generation [53]. Amyloid aggregates further stimulate astrocytes to secrete exosomes enriched in ceramide and prostate apoptosis response 4 (PAR-4), which are subsequently internalized by neighboring astrocytes and trigger apoptotic signaling, thereby amplifying glial dysfunction [54]. These findings highlight a reciprocal interplay whereby amyloid pathology and ADEs mutually reinforce neurodegenerative processes.

Microglia-derived exosomes similarly contribute to the dissemination of tau and amyloid-β pathology across brain regions. Multi-omics profiling of microglial exosomes reveals reduced expression of homeostatic markers (P2RY12, TMEM119), increased disease-associated microglial signatures, and altered lipid composition in AD models and patient tissue [55,56]. Experimental depletion of microglia or pharmacological inhibition of microglial exosome secretion markedly suppresses tau propagation, highlighting microglial exosomal pathways as tractable therapeutic targets [60]. Given that glia-derived EVs can be present in both central and peripheral samples, differences in isolation strategies, cellular enrichment, and disease context may contribute to variability across studies, thereby complicating direct comparisons and clinical interpretation.

### 2.6. Application Progress of EV-Associated Biomarkers in AD

EV-associated Aβ42, total tau, and phosphorylated tau species (p-tau181, p-tau217, and p-tau231) are among the most extensively studied biomarker candidates and have been consistently reported to correlate with CSF measures, cognitive performance, and longitudinal disease progression across multiple cohorts. Across different EV sources—including peripheral blood, neuron-enriched EVs, and CSF-derived EVs—these core AD-related markers appear to provide convergent yet context-dependent information, reflecting distinct aspects of disease biology, including systemic responses, neuronal pathology, and CNS-specific processes. Recent analytical platforms, including a drop-shaped porous microfluidic chip enabling multiplexed, high-sensitivity detection and a Ni@Pt nanoparticle-based colorimetric system for rapid plasma Aβ42 quantification, have further improved analytical feasibility by reducing sample volume and assay time [61,62]. Nevertheless, despite their promising performance in discovery and early validation studies, these EV-associated markers remain at a preclinical-to-translational stage. Among these, EV-associated Aβ and tau species represent the most advanced candidates, having undergone multi-cohort validation, whereas many other EV-derived molecules remain in early discovery or validation phases.

Complement proteins have been reported to be reproducibly altered in EVs from AD patients, suggesting sustained immune activation, whereas synaptic proteins are frequently observed to be reduced in neuron-enriched EV fractions, consistent with early synaptic dysfunction. These changes have been observed in several populations and show prediction capability years prior to the onset of cognitive impairment, but they are still in the validation stage because they require additional cross-population testing and standardization of isolation methods.

Additional candidates, including AACT, zyxin, and TDP-43, as well as astrocyte-associated cargos such as BACE1, sAPPβ, ceramide, and PAR-4, have been identified through proteomic profiling and mechanistic studies. However, current evidence for these molecules is largely confined to discovery-level or experimental datasets, and reported clinical associations remain variable or limited. Accordingly, these markers should be considered exploratory, pending systematic validation in independent human cohorts.

EVs isolated from urine and brain tissue in AD mouse models have been reported to exhibit early alterations in clusterin, annexin 2, neuraminidase-1, lipid composition, and plaque-associated accumulation. Although these findings provide mechanistic insight into EV-associated pathology, they remain restricted to preclinical models and have not yet been translated into validated human biomarkers.

Although EV-based biomarkers may provide multiplexed molecular information and mechanistic insight into AD pathophysiology, they currently do not outperform established clinical biomarkers such as plasma p-tau217 or CSF Aβ42/40 ratios for first-line diagnosis. Such variation could be due to biological diversity and experimental variability among studies. On the contrary, EV biomarkers seem well-suited as auxiliary tools in disease stratification, mechanistic endophenotyping, assessment of therapeutic efficacy, and cell-type-specific disease pathology. In summary, EV biomarkers range from those that are well-characterized and close to being clinically implemented to those that are less characterized and need further investigation. It is important to classify the different types of biomarkers to interpret their clinical applicability.

### 2.7. Bioinformatic Analysis of Differentially Expressed miRNAs in EVs from Patients with AD

Apart from changes in protein content, EV-related non-coding RNA (ncRNA), especially microRNAs (miRNAs), have been shown to be dysregulated in AD and serve as promising candidates as biomarkers. In all EVs isolated from various biological origins (such as plasma, neuron-enriched extracts, CSF, and urine), the miRNA modifications exhibit similarities and differences depending on their biological origin, indicating the heterogeneous nature of the EV signaling pathway in AD. These patterns may capture distinct aspects of disease biology, including systemic responses, neuronal dysfunction, and CNS-specific pathological processes.

Multiple studies have reported increased levels of miR-106a-5p, miR-16-5p, miR-25-3p, miR-30b-5p, miR-92a-3p, miR-223-3p, and miR-451a in plasma EVs from AD patients, with miR-30b-5p and miR-223-3p showing correlations with Aβ42 and tau levels [33,34]. Combinatorial panels, such as miR-135a, miR-193b, and miR-384, have been reported to improve diagnostic performance compared with individual miRNAs [35]. In neuron-enriched EV fractions, elevated levels of miR-29c-5p, miR-143-3p, miR-335-5p, miR-485-5p, miR-29c-3p, and miR-384 have been consistently described, including in familial AD mutation carriers [44,45,46]. CSF EVs have been reported to exhibit increased levels of miR-125b-5p, miR-27a-3p, miR-30a-5p, and miR-34c, as well as specific piRNAs (piR-019949 and piR-020364) [48,49]. In animal models, urinary EVs have been observed to show upregulation of miR-196b-5p, miR-34a-5p, and miR-376b-3p, suggesting potential involvement in early pathological changes [51]. Together, these findings suggest that while specific miRNA signatures vary across EV sources, a subset of dysregulated miRNAs may converge on key AD-related pathways, including amyloid processing, tau pathology, and neuroinflammation.

Several synaptic and neuroprotective miRNAs have been reported to be reduced in AD-associated EVs. In plasma EVs, miR-22-3p and miR-378a-3p are decreased [34]. Neuron-enriched EV fractions show reduced levels of miR-138-5p, miR-342-3p, miR-132-3p, and miR-212-3p, which have been associated with impaired synaptic plasticity [44,47]. In CSF EVs, miR-451a, miR-605-5p, and piR-019324 have been reported to be downregulated [48,49].

To compile the studies included in Table 1, a structured literature search was performed using PubMed and Web of Science (2015–2025) with the terms “Alzheimer’s disease,” “extracellular vesicle,” “exosome,” “miRNA,” and “differential expression.” Eligible studies analyzed EV-associated miRNA profiles in AD and control samples, reported the biological source of the EVs (e.g., plasma, CSF, urine, neuron-enriched fractions), employed validated quantification methods (RNA sequencing or qPCR), and provided statistically significant differential expression data. Differentially expressed miRNAs were integrated and analyzed using the miRNet platform, and DisGeNET mapping identified 31 AD-associated target genes among the upregulated miRNA set (Table 2). These included BACE1, glycogen synthase kinase 3β (GSK3β), and tau, which are implicated in the axis underlying amyloid-β deposition and neurofibrillary tangle formation [63,64,65], as well as triggering receptor expressed on myeloid cells 2 (TREM2), ABCA7, CD33, and clusterin, which are involved in microglial-mediated amyloid clearance and neuroinflammation [66,67,68,69]. DisGeNET analysis of downregulated miRNAs yielded eight AD-related target genes, including enolase 1, visinin-like protein 1, and acetylcholinesterase, which are associated with metabolic stress, neuronal injury, and cholinergic dysfunction, respectively [70,71,72].

**Figure 3:**
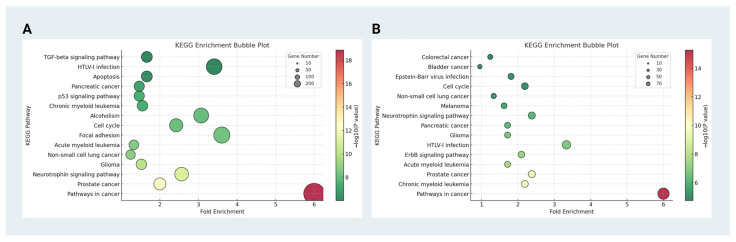
KEGG enrichment analysis of target genes for differentially expressed miRNAs. Bubble plots illustrating the KEGG pathway enrichment results for (**A**) upregulated and (**B**) downregulated miRNA target genes identified from exosomal profiles in AD patients. The *x*-axis represents the fold enrichment, and the size of each bubble corresponds to the number of genes enriched in the pathway. The color gradient indicates statistical significance, with warmer colors representing smaller *p*-values.

KEGG enrichment analysis suggested that predicted targets of upregulated miRNAs are enriched in cancer-related and apoptosis-associated pathways, whereas downregulated miRNA targets are associated with overlapping oncogenic, neurodevelopmental, and survival signaling pathways (Figure 3). The prominence of cancer-related pathways in both datasets likely reflects not only shared dysregulation of cell-cycle control, metabolic stress responses, and survival signaling, but also potential biases in pathway annotation and database composition, as EV research has been extensively studied in cancer contexts. Therefore, these pathway enrichments should be interpreted with caution and considered indicative of broadly dysregulated biological processes rather than cancer-specific mechanisms. These pathway enrichments further support the notion that EV-associated miRNAs may regulate interconnected biological processes rather than acting as isolated disease markers.

All these findings taken together indicate that the miRNAs associated with EVs could converge on important processes linked to AD, such as Aβ processing, tauopathy, inflammation of the nervous system, metabolic stress, and impaired synapses. Still, since target genes are predicted using computational methods and manually curated data, such networks should be regarded as hypothesis-generating, not causal. The functional role of these miRNA-target interactions will need to be validated through studies of patient-derived models of neurons and glial cells. However, variations in the source of EVs, detection methods for miRNAs, normalization strategies, and the nature of the cohort have been shown to cause heterogeneity among the studies. These issues would need to be sorted out if standardized biomarkers associated with EV miRNAs are to be developed.

## 3. EV-Mediated Spread of Pathological Proteins

The EV-mediated transport of pathogenic proteins, especially amyloid-β and tau proteins, is increasingly seen as an essential pathway of AD development. Accumulating evidence indicates that EVs can facilitate the intercellular transfer of misfolded or aggregation-prone protein species, and this process has been proposed to resemble a prion-like mode of propagation [73]. However, current evidence primarily supports the role of EVs as carriers of seeding-competent protein assemblies, whereas direct demonstration of conformational templating occurring within the same EV populations remains limited. Instead, protein aggregation is more likely to occur after EV uptake in recipient cells, where internalized cargo can initiate downstream pathological processes.

### 3.1. Aβ Propagation via EVs

Although Aβ plaques accumulate extracellularly, the most neurotoxic isoform, Aβ42, originates intracellularly and is trafficked through the endosomal–lysosomal system [74,75]. Neuronal accumulation of Aβ42 alters MVB morphology, leading to MVB enlargement and fibrillar aggregate formation, as observed in both AD model mice and wild-type mice administered Aβ42 [76]. ESCRT proteins, which are essential for MVB biogenesis, colocalize with Aβ plaques, indicating direct involvement of the endosomal pathway in Aβ aggregation. Impaired MVB turnover caused by VPS4A dysfunction or lysosomal defects significantly increases intracellular Aβ42 [76].

**Figure 4:**
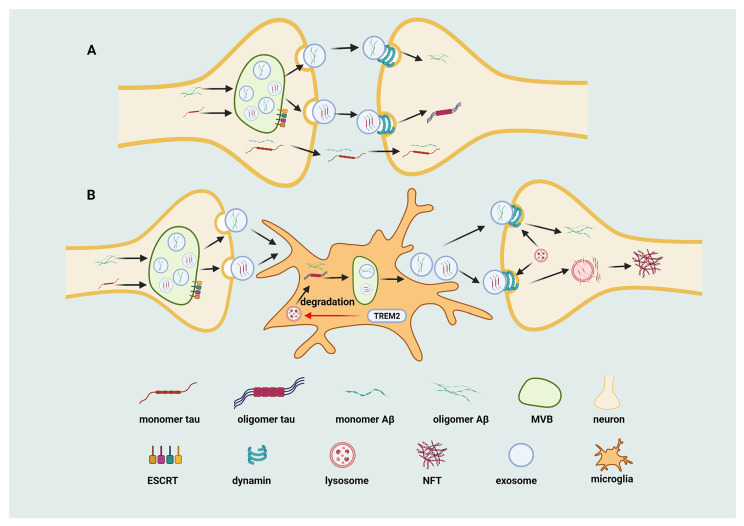
EV-mediated transmission of pathological proteins in AD: (**A**) Neuron-to-neuron transmission of pathological proteins. Intracellular Aβ and tau proteins are sorted into MVBs via the ESCRT machinery and released in EVs enriched with oAβ and tau. These EVs are internalized by adjacent neurons via dynamin-dependent endocytosis, leading to the release of toxic proteins and their intracellular aggregation. In parallel, soluble Aβ and tau species can be directly transmitted across synapses without vesicular encapsulation, contributing to rapid local spread and pathological seeding in recipient neurons. (**B**) Microglia mediate long-range propagation of tau pathology through TREM2-regulated exosomal trafficking. Neuron-derived tau-containing EVs are internalized by microglia, where tau is either degraded via the lysosomal pathway or rerouted into preexosomal compartments and resecreted. Loss of TREM2 impairs lysosomal degradation and enhances secretion, facilitating tau transfer to secondary neurons and promoting NFT formation. TREM2 also promotes exosome-mediated Aβ clearance by microglia, underscoring its dual regulatory role in modulating both tau and Aβ pathologies.

EVs released from AD brain tissue are enriched in toxic oligomeric Aβ (oAβ), predominantly within the vesicle lumen, and these EVs are efficiently internalized by neurons, leading to propagation of neurotoxicity [77]. Genetic or pharmacological disruption of ESCRT components such as TSG101 or VPS4A markedly reduces EV release and suppresses oAβ transfer between neurons. In parallel, inhibition of dynamin-dependent endocytosis attenuates neuronal uptake of EVs, indicating that both EV biogenesis and uptake pathways represent potential therapeutic targets for limiting Aβ spread in AD [77] (Figure 4).

Importantly, while there is a growing body of data in favor of the theory that Aβ42 accumulation within neurons and MVB defects result in EV loading of Aβ oligomers, several issues remain unresolved. For example, while much information comes from in vitro models and analysis of postmortem brains, little is known about the dynamics of Aβ spreading via EVs in living subjects. Importantly, recent research utilizing sophisticated imaging and electrophysiology techniques has shed light on stimulus-driven dynamics of EV secretion from neurons, including live visualization of multivesicular body fusion with the plasma membrane [78]. This information offers valuable insights into the mechanisms regulating EV secretion. Yet, our understanding of the temporal control of EV-mediated Aβ spreading in vivo, especially in relation to AD pathogenesis, is incomplete. Additionally, the exact causal role of EVs in Aβ dissemination is not fully clarified; namely, it is not clear whether EVs actively drive Aβ dissemination or rather play a passive role of secondary agents amplifying the initial aggregation of Aβ. In addition, the relative importance of EV-associated and soluble Aβ oligomers is yet to be quantitatively assessed.

Overall, the available data suggest a facilitative rather than causative contribution of EVs to Aβ spreading; however, it is possible that the nature of their contribution changes depending on the state of the disease and interaction with other modes of propagation. In future research, attention should be paid to the comparative assessment of Aβ species inside EVs and freely circulating in the brain fluid. In addition, systematic comparison of MVB/ESCRT-dependent pathways with alternative Aβ release mechanisms will be essential to establish whether targeting EV biogenesis, cargo loading, or uptake represents a viable strategy for halting Aβ propagation in AD.

### 3.2. Tau Propagation via EVs

Unlike Aβ, tau is predominantly an intracellular protein but can be released into both central and peripheral compartments. EV-associated tau derived from neurons represents only a small fraction of total extracellular tau and typically exhibits relatively low phosphorylation levels [79,80]. Nevertheless, neuronal activity enhances its release, and tau has been detected within the lumen of EVs [81]. EV-associated tau supports intercellular transfer between neurons as well as between neurons and microglia [80,82]. Consistent with this, EV-associated tau has been shown to possess seeding activity and can induce aggregation in recipient cells, supporting its role in the propagation of tau pathology [83]. Synaptic structures are critical for neuron-to-neuron transfer, whereas microglia-derived EVs facilitate tau dissemination between nonadjacent neurons in a process regulated by TREM2 [84]. Deletion of TREM2 enhances microglial EV-mediated tau transfer, whereas TREM2 also promotes EV secretion that supports Aβ clearance, underscoring its dual role in regulating both Aβ and tau pathology [84,85,86] (Figure 4).

EV-mediated tau propagation is further shaped by lysosomal processing. Tau-containing EVs internalized by recipient cells traffic to endolysosomal compartments, where acidic conditions promote tau escape into the cytosol and facilitate seeding and aggregation, although whether such seeding is initiated within EVs or predominantly after release into recipient cells remains to be fully clarified [87]. Inhibition of lysosome biogenesis or alkalinization of lysosomes attenuates this process.

Importantly, although EV-associated tau transfer is well documented, soluble oligomeric tau and synaptically released tau also contribute to intercellular spread [88,89,90]. The relative contribution of vesicular versus non-vesicular pathways remains unclear and is likely to vary across disease stages and levels of neuronal activity. Another unresolved issue is the basis of selective neuronal vulnerability, as entorhinal and hippocampal neurons are among the earliest affected populations in AD [91]. Candidate surface molecules, including tetraspanins and integrins, have been proposed to mediate selective EV targeting [92], but direct in vivo evidence remains limited. Future studies should therefore prioritize quantitative in vivo mapping of tau transfer routes and mechanistic dissection of microglial and synaptic EV pathways to clarify how tau dissemination drives AD progression and to identify context-specific therapeutic targets. Overall, EV-associated tau contributes to intercellular spread, but whether EVs serve as initiating factors or secondary amplifiers of pathology remains unresolved.

## 4. Regulation of AD Progression by EVs from Distinct Cellular Origins

### 4.1. Neuron-Derived EVs

EVs released from primary neurons promote conformational changes in soluble extracellular Aβ, driving its conversion into relatively less toxic amyloid protofibrils [93]. This activity is closely associated with glycosphingolipids on the EV membrane, particularly the ganglioside GM1, which is enriched in endosome-derived EVs. Inhibition of GM1, either by blocking its binding sites with cholera toxin B subunit or by cleaving the β-glycosidic bond between the GM1 sugar chain and ceramide using endoglycoceramidase, suppresses EV-mediated Aβ fibrillization. In addition, phosphatidylserine exposed on the EV surface facilitates microglial uptake and enhances Aβ clearance. Consistently, oral administration of phytoceramides in AD mice increases neuronal EV release and promotes EV-dependent Aβ clearance, resulting in improved cognitive function [94].

**Figure 5:**
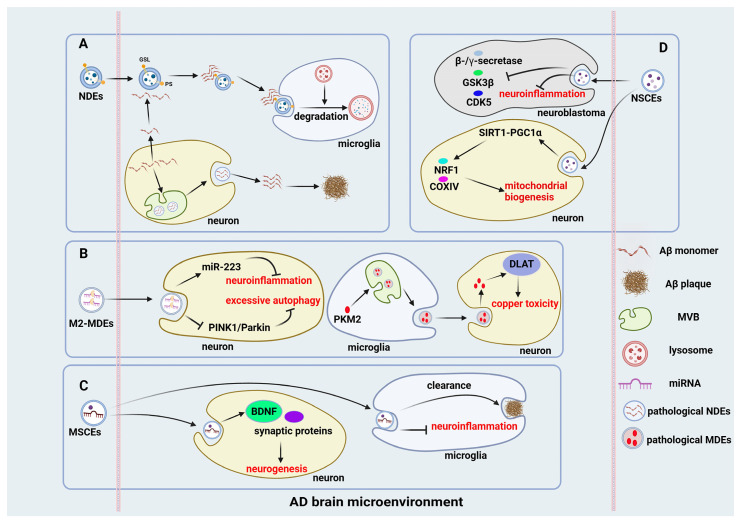
Regulation of AD progression by EVs from different cellular origins: (**A**) In the AD brain, pathological neuron-derived EVs (NDEs) promote Aβ aggregation by transporting monomers to extracellular sites. In contrast, primary NDEs convert soluble Aβ into nontoxic protofibrils via glycosphingolipids, whereas exosomal phosphatidylserine aids in microglial uptake and Aβ clearance. (**B**) M2 microglia-derived EVs (M2-MDEs) enriched with miR-223 attenuate neuroinflammation and inhibit excessive autophagy via the PINK1/Parkin pathway. In contrast, pathological MDEs carrying PKM2, which is modulated by hnRNPA2B1 and Sepp1, exacerbate copper-induced neurotoxicity through the DLAT pathway in recipient neurons. (**C**) Intranasally or intravenously delivered mesenchymal stem cell-derived EVs (MSCEs) selectively target brain lesions in AD, facilitating microglial anti-inflammatory polarization, enhancing Aβ phagocytosis and promoting neurogenesis. (**D**) Neural stem cell-derived EVs (NSCEs) exhibit neuroprotective and neurogenic properties by reducing Aβ and p-tau accumulation via β-/γ-secretase, GSK3β, and CDK5 regulation. NSC-Exos suppress neuroinflammation, increase cell survival, and restore mitochondrial function through SIRT1-PGC1α signaling.

While neuronal EVs facilitate microglial clearance of Aβ, they can also transport Aβ to extracellular aggregation sites. In APP^NL–F mouse brains, EV abundance is increased, and these EVs colocalize with Aβ plaques [95]. Chronic inhibition of neutral sphingomyelinase 2 (nSMase2) reduces ceramide production, disrupts glycosphingolipid metabolism, and suppresses neuronal EV release, thereby attenuating Aβ dispersion and plaque formation. Thus, neuronal EVs exert dual and context-dependent effects in AD, promoting both Aβ clearance and extracellular aggregation. Precise regulation of EV biogenesis and lipid composition may therefore represent a viable strategy for modulating Aβ pathology in AD (Figure 5).

### 4.2. Microglia-Derived EVs

In vitro and in vivo studies have shown that EVs released from M2-polarized microglia are enriched in miR-223, which attenuates neuronal injury in AD cellular models and improves cognitive performance in AD mice [96]. Microglial miR-223 is selectively sorted into endosome-derived EVs by the RNA-binding protein Y-box-binding protein 1 (YB-1), and inhibition of YB-1 disrupts EV-mediated miR-223 transfer, thereby abolishing its neuroprotective effects in both cellular and animal models of AD. In parallel, EVs derived from M2 microglia also alleviate neuronal injury and mitochondrial dysfunction by suppressing excessive autophagy through the PINK1/Parkin pathway [97].

In contrast, EVs released from microglia in AD models can also exert deleterious effects. Microglia-derived EVs in AD mice are highly enriched in pyruvate kinase M2 (PKM2), which, upon neuronal uptake, interacts with intracellular targets to increase dihydrolipoamide S-acetyltransferase expression and exacerbate copper-dependent neuronal death [98]. The cleavage, trafficking, and EV packaging of PKM2 are regulated by the RNA-binding protein hnRNPA2B1, and inhibition of microglial EV-mediated PKM2 transfer mitigates neuronal loss and cognitive impairment in AD model mice.

Microglia associated with neurodegeneration exhibit intrinsic regulatory programs that govern EV biogenesis and secretion. Silencing selenoprotein P (Sepp1) increases lipid metabolic stress and lysosomal activity in microglia, thereby suppressing the formation and release of microglia-derived EVs [99]. By limiting EV secretion, Sepp1 deficiency may attenuate the progression of AD pathology.

Astrocytes and microglia share common regulatory features in EV biogenesis and secretion, including disease-associated remodeling and Sepp1-dependent control [99]. Transcriptomic profiling of EV-associated RNAs from astrocytes and microglia has revealed enrichment of hub genes, including ubiquitin B and heat shock protein family A members, which are implicated in proteostasis and neurodegenerative processes [100].

### 4.3. Mesenchymal Stem Cell-Derived EVs

Mesenchymal stem cells (MSCs) are widely used for EV-based therapeutic strategies owing to their availability, low immunogenicity, high secretory capacity, and favorable safety profile. EVs released from MSCs (MSC-EVs) can be engineered to incorporate glucose-coated gold nanoparticles, enabling EV-associated nanoprobes to accumulate in brain lesion regions in models of Parkinson’s disease, AD, and stroke following intranasal administration [101,102]. Gold nanoparticle-based neuroimaging has been used to visualize the biodistribution and brain accumulation of MSC-EVs, suggesting preferential localization to sites of neurodegeneration.

Stereotactic brain injection of MSCs or MSC-EVs alleviates Aβ-induced cognitive impairment and enhances neurogenesis in mice [103]. Fluorescent tracing studies show that intracerebroventricularly delivered MSC-EVs are internalized by astrocytes and neurons [104]. Intranasal administration of MSC-EVs promotes anti-inflammatory polarization of microglia in AD models, thereby exerting immunomodulatory and neuroprotective effects [105]. In addition, MSC-EV treatment reduces the colocalization of glial fibrillary acidic protein with Aβ plaques, indicating attenuation of astrocyte activation and neuroinflammatory responses [106]. EVs derived from bone marrow MSCs and adipose-derived MSCs regulate glial activity, autophagy, apoptosis, and inflammatory signaling through ncRNAs and protein cargos, leading to upregulation of neuroprotective pathways and improved cognitive and pathological outcomes in AD model mice [107,108,109,110].

EVs derived from human umbilical cord blood MSCs (hUCB-MSC-EVs) similarly exhibit therapeutic potential in AD. When delivered intravenously or intranasally, hUCB-MSC-EVs enhance Aβ clearance, promote anti-inflammatory microglial polarization, and increase the expression of hippocampal plasticity-associated proteins, resulting in improved learning and memory in AD model rodents [111,112]. Declining cerebral glucose metabolism is a hallmark of neuronal dysfunction in AD [113], and positron emission tomography using [^18F]FDG has shown that hUCB-MSC-EV treatment partially restores brain glucose utilization in AD mice [114]. Although intracerebroventricular transplantation of hUCB-MSCs is currently being evaluated in phase I clinical trials, this approach is associated with transient inflammatory adverse events, including fever, headache, nausea, and vomiting [115,116]. These safety concerns underscore the potential advantages of using hUCB-MSC-derived EVs as a cell-free alternative for AD therapy.

### 4.4. Neural Stem Cell-Derived EVs

Neural stem cell-derived EVs (NSC-EVs) represent a promising class of therapeutic vesicles owing to their relatively homogeneous cellular origin and intrinsic neural lineage-associated signaling properties. Compared with EVs from peripheral MSCs, NSC-EVs may provide enhanced neuroprotective and neurogenic effects in the context of AD. In vitro studies demonstrate that NSC-EVs reduce the accumulation of Aβ and phosphorylated tau by modulating the activity of β- and γ-secretases, GSK3β, and cyclin-dependent kinase 5 (CDK5) [117]. In parallel, NSC-EVs suppress neuroinflammatory pathways and pro-inflammatory mediators while enhancing neuronal survival in SH-SY5Y cells, thereby mitigating AD-associated cellular pathology.

NSC-EVs also activate the SIRT1–PGC1α signaling axis, leading to increased expression of nuclear respiratory factor 1 and cytochrome c oxidase subunit IV, enhanced mitochondrial biogenesis, and reduced astrocyte activation [118]. These effects contribute to the reversal of mitochondrial dysfunction and abnormal spatial distribution of mitochondria in AD model mice. Furthermore, hippocampal NSC-EVs are taken up by neurons and prevent oAβ-induced memory deficits by restoring long-term potentiation [119]. At the synaptic level, NSC-EVs protect against oAβ-induced synaptic injury by reducing synapse–Aβ binding and enhancing calcium/calmodulin-dependent protein kinase II phosphorylation, thereby preserving synaptic plasticity.

## 5. Dynamic Regulation of EV Biology by the Cellular Microenvironment

The cellular microenvironment profoundly shapes EV biogenesis, cargo composition, and functional activity. While many of these regulatory mechanisms have been established in general EV biology, their direct relevance to AD varies depending on the context and experimental system. In this section, we distinguish between mechanisms that have been directly demonstrated in AD models or patient-derived systems and those inferred from broader EV studies that may be relevant to AD pathophysiology. These microenvironmental factors—including inflammation, lipid metabolism, hypoxia, and physical cues—are likely to influence EV-mediated intercellular communication in AD, although the extent of their disease-specific contributions remains to be fully established.

### 5.1. Inflammatory Cues

Neuroinflammation is a central feature of AD, and immune-mediated regulation of microglia critically determines both inflammatory burden and Aβ clearance. EVs released under inflammatory stimulation exert potent immunomodulatory effects on neural and glial networks. Exposure of human immortalized neural progenitor cells to IL-6 markedly increases the levels of hsa-miR-135a-3p, hsa-miR-6790-3p, and hsa-miR-11399 in their secreted EVs. Upon EV uptake, these miRNAs—normally expressed at low levels in microglia and astrocytes—suppress mRNAs involved in neuroregeneration, thereby limiting neural repair capacity [120].

Treatment of BV2 microglia with IL-10 or TGF-β reduces chaperonin-containing TCP-1 complex proteins and increases proteasome-associated proteins in EV cargo, whereas lipopolysaccharide (LPS) stimulation induces broad remodeling of EV-associated mRNA and miRNA profiles that propagate proinflammatory signaling to recipient cells [121]. In response to inflammatory activation, microglia also secrete high levels of neuraminidase-3, which is transferred to neurons via EVs, leading to disruption of the neuronal glycocalyx and reduced network connectivity [122].

Astrocyte-derived EVs similarly undergo inflammatory polarization. EVs released following IL-1β stimulation carry protein cargos that promote peripheral immune activation, whereas EVs induced by IL-10 are enriched in factors involved in axonal guidance, gap junction formation, and CREB signaling. Consistently, exposure of primary cortical neurons to IL-1β-conditioned astrocyte EVs markedly suppresses neuronal activity [123]. Astrocytes exposed to TNFα, LPS, and z-VAD (TLZ), a necroptotic inflammatory stimulus, secrete EVs with elevated levels of precursor brain-derived neurotrophic factor (BDNF), which promotes neuronal apoptosis [124].

Peripheral macrophage-derived EVs generated following exposure to LPS or interferon-γ can also activate microglia and astrocytes in the brain, an effect associated with increased EV-associated miR-155-5p [125]. In contrast, EVs released from bone marrow-derived mesenchymal stem cells (BMSCs) under IL-1β stimulation suppress LPS-induced astrocyte proliferation and inflammatory responses, reflecting the intrinsic immunoregulatory properties of BMSCs [126]. Together, these findings highlight the strong cell type- and stimulus-dependent plasticity of EV cargo and function, underscoring the importance of microenvironmental context in determining whether EVs exacerbate or attenuate AD-related neuroinflammation.

### 5.2. Cholesterol Exposure

The apolipoprotein E4 (ApoE4) variant is the strongest genetic risk factor for sporadic AD, and ApoE4-expressing astrocytes exhibit impaired Aβ uptake and pathological cholesterol accumulation [127]. ApoE4-driven lysosomal cholesterol retention disrupts mitochondrial homeostasis and oxidative phosphorylation in astrocytes [128]. Astrocytes derived from ApoE-deficient mice display markedly reduced EV release and increased extracellular cholesterol levels, and direct cholesterol loading of wild-type astrocytes similarly suppresses EV secretion [129].

Pharmacological inhibition of cholesterol trafficking further induces cholesterol accumulation in astrocytes, resulting in EVs enriched with amyloid precursor protein (APP), APP C-terminal fragments, soluble APP, secretases, and Aβ1–40, while simultaneously impairing the viability of recipient neurons [130]. These observations indicate that astrocytic cholesterol homeostasis critically regulates EV biogenesis and cargo loading, thereby influencing the spread of amyloidogenic and metabolic stress signals in AD.

Together, these findings support a model in which cholesterol dysregulation reshapes astrocyte-derived EV pathways and mitochondrial function, amplifying neurodegenerative cascades. Targeting EV-mediated lipid trafficking and organelle stress responses, therefore, represents a mechanistically grounded strategy for modulating AD progression. Given the established role of ApoE4 and lipid dysregulation in AD, these findings provide relatively direct relevance to AD pathophysiology.

### 5.3. Oxygen–Glucose Deprivation and Hypoxic Conditioning

Although hypoxia-related EV regulation has been extensively studied in broader neurobiological contexts, its direct role in AD remains partially defined. Oxygen–glucose deprivation (OGD) profoundly alters astrocyte viability and EV signaling. EVs released from astrocytes under OGD display distinct miRNA signatures, and RNA sequencing shows that differentially expressed miRNAs and their target genes are enriched in pathways related to stress responses, neuroprotection, and signaling relevant to AD progression [131]. In AD, oligodendrocyte progenitor cell (OPC) migration and regeneration are impaired, leading to reduced oligodendrocyte numbers and compromised myelin integrity [132,133]. Modulation of astrocyte-derived EVs generated under moderate OGD conditions promotes OPC differentiation, migration, and myelination, whereas severe deprivation suppresses OPC proliferation [134]. Astrocyte-derived EVs produced under ischemic–hypoxic conditions also show increased prion protein levels, which protect neurons against oxidative stress-induced injury [135], suggesting that OGD-programmed astrocyte EVs can exert context-dependent neuroprotective effects in AD.

Chronic intermittent hypoxia exacerbates tau pathology and memory impairment in AD mice [136]. In intermittent hypoxia models, microglia-derived EVs suppress neuroinflammation and improve cognitive performance by inhibiting neuronal NLRP3 inflammasome activation [137]. These EVs exhibit elevated miR-146a-5p, which targets HIF1α and reduces mitochondrial reactive oxygen species, thereby attenuating inflammatory signaling.

Hypoxic preconditioning also enhances the therapeutic potential of mesenchymal stem cell-derived EVs. EVs from adipose-derived MSCs exposed to hypoxia mitigate hippocampal neuronal damage, suppress neuroinflammation, and improve cognitive function in AD models. These effects are mediated in part by circ-Epc1, which regulates the circ-Epc1/miR-770-3p/TREM2 axis [138]. Similarly, EVs from hypoxia-preconditioned bone marrow-derived MSCs display superior neuroprotective efficacy compared with normoxic MSC-EVs. Intravenous delivery of these EVs improves learning and memory, increases neuroprotective protein expression, and reduces neuroinflammatory mediators in AD model mice, with miR-21 contributing substantially to these benefits [139].

In contrast to glial and MSC-derived EVs, EVs released from neurons under OGD and reperfusion exert detrimental effects on neighboring neurons. Compared with normoxic neuronal EVs, hypoxia-conditioned neuronal EVs markedly reduce cortical neuron viability and impair neurite outgrowth [140]. Their miRNA cargo is enriched in regulators of apoptosis, cell survival, and neuronal signaling. Notably, hypoxia-induced neuronal EVs show a time-dependent increase in miR-21a-5p that precedes its upregulation in tissues and cells [141]. Although the functional relevance of EV-associated miR-21a-5p in AD remains unresolved, it represents a potential target for further mechanistic investigation.

### 5.4. Physical Factor Regulation

Physical factors have been shown to modulate EV biology across multiple experimental systems, although their direct relevance to AD remains limited or context-dependent. Physical forces modulate cellular signaling, metabolism, and stress responses, thereby reshaping EV biogenesis, cargo composition, and biological activity. In astrocytes, intermittent ultrasound stimulation enhances MVB formation and increases the release of astrocyte-derived EVs. Co-treatment of SH-SY5Y cells with ultrasound-conditioned astrocyte EVs and Aβ promotes intracellular colocalization, limits Aβ uptake, and reduces cytotoxicity [142]. In APP/PS1 mice, focused ultrasound combined with microbubbles to transiently open the blood–brain barrier, followed by intravenous delivery of ultrasound-conditioned astrocyte EVs, enhances Aβ plaque clearance, demonstrating that physical modulation of EV production can be leveraged to augment therapeutic efficacy.

Photobiomodulation has also emerged as a promising physical intervention in AD, particularly through its effects on microglia [143,144,145]. BV2 microglia exposed to 1070 nm light reverse LPS-induced M1 polarization and release EVs that protect neurons from inflammatory injury and Aβ accumulation, promote neurite outgrowth, and improve cognitive performance in AD models following intranasal administration [146]. Mechanistically, EVs generated by photobiomodulated microglia are enriched in miR-7670-3p, which suppresses activating transcription factor 6, thereby attenuating neuroinflammation, Aβ deposition, and cognitive deficits in AD models.

EVs generated in response to other physical stimuli also exhibit potent bioactivity. For example, hypothermia-conditioned microglial EVs enhance neurite outgrowth and synaptic recovery after traumatic brain injury, whereas EVs produced under high-pressure stress exacerbate retinal neuroinflammation [147,148]. Although these paradigms have not yet been tested in AD models, they underscore the strong sensitivity of EV phenotypes to physical microenvironmental cues and highlight opportunities for engineering EV-based interventions for neurodegenerative disease. Although these paradigms have not yet been systematically validated in AD models, they underscore the strong sensitivity of EV phenotypes to physical microenvironmental cues and suggest potential avenues for future investigation in AD.

## 6. EV Bioengineering for Therapeutic Applications in AD

EVs are useful nanocarriers for delivering therapies because of their biocompatibility, natural uptake into cells, and ability to carry bioactive payloads. As compared with existing drug-delivery systems such as adeno-associated virus (AAV)-based vectors and lipid-based nanoparticles (LNPs), EVs have several unique features, including reduced immunogenicity, the potential for repeated dosing, and intrinsic biological targeting properties. However, they also face challenges related to biodistribution, scalability, and in vivo stability. Bioengineering strategies, including nucleic acid loading, surface functionalization, and targeting ligand incorporation, have substantially expanded the translational potential of EVs. This section summarizes recent advances in EV bioengineering aimed at optimizing therapeutic efficacy in AD (Table 3), with a later section providing a detailed comparison of EVs with conventional delivery platforms.

### 6.1. miRNA-Based EV Engineering

Within this context, EV engineering strategies can be interpreted relative to existing delivery platforms, with distinct advantages and limitations compared with viral and synthetic systems. Noncoding RNAs, particularly microRNAs, are among the most biologically active EV cargos and therefore represent major targets for EV-based genetic engineering. Dendritic cells transfected with miR-29b-2 using viral vectors secrete EVs that suppress presenilin 1 expression and reduce oAβ formation in AD cellular and animal models [149]. In parallel, co-engineering EVs with cholecystokinin/somatostatin peptides and CD47 enhances brain accumulation while reducing phagocytic clearance following systemic administration.

Similarly, transfection of BMSCs and HEK-293T cells with miR-29a or miR-29b precursor sequences yields EVs enriched in miR-29. When administered to AD model mice, these engineered EVs inhibit BACE1, attenuate Aβ pathology, and improve cognitive performance [150]. As noted above, miR-22 is reduced in EVs isolated from the peripheral blood of AD patients, highlighting its potential as a therapeutic target [34]. Engineering adipose-derived MSCs to overexpress miR-22 produces EVs enriched in this miRNA, and systemic delivery of these EVs to APP/PS1 mice results in greater suppression of neuroinflammation and improved behavioral outcomes compared with unmodified EVs [151].

Collectively, these studies demonstrate that EVs engineered to carry disease-modifying miRNAs can attenuate Aβ pathology, modulate neuroinflammation, and enhance cognitive function in AD models, supporting EV-based RNA delivery as a promising therapeutic strategy.

### 6.2. Bioactive Molecule Loading

EVs can be directly loaded or bioengineered to carry small molecules, peptides, and natural products to modulate AD pathology. Bone marrow-derived MSC EVs functionalized with rabies virus glycoprotein (RVG) peptides display enhanced accumulation in the CNS and outperform unmodified EVs in reducing Aβ plaque burden, suppressing neuroinflammation, and improving cognitive function in AD models [152].

Compared with free coenzyme Q10 (CoQ10) or unmodified EVs, intraperitoneal delivery of adipose-derived MSC EVs loaded with CoQ10 provides superior neuroprotection and cognitive benefit in AD model rats, accompanied by increased hippocampal expression of BDNF and SOX2 [153]. Similarly, bone marrow MSC EVs loaded with olesoxime and resveratrol via electroporation attenuate Aβ-induced oxidative stress and apoptosis in SH-SY5Y cells and improve learning and memory in APP/PS1 mice following systemic administration [154]. EVs derived from microglia and loaded with the plant alkaloids berberine and palmatine enhance drug delivery to the brain and reduce neuroinflammation and cognitive impairment in APP/PS1 mice, although residual peripheral drug retention highlights current limitations in CNS-selective targeting [155].

In addition to direct cargo loading, EV composition can be modified by altering the cellular microenvironment. Treatment of astrocytes with catalpol and tetramethylpyrazine induces the release of EVs with elevated CDK5 content. Moderate CDK5 activity promotes EV-mediated neuroprotection and axonal plasticity through signal transducer and activator of transcription 3 phosphorylation in AD models, whereas excessive CDK5 activation exacerbates tau hyperphosphorylation and neurodegeneration [156]. Consistently, intravenous administration of plasma-derived EVs loaded with quercetin suppresses CDK5-mediated tau phosphorylation and neurofibrillary tangle formation, thereby mitigating AD pathology in mice [157]. These findings illustrate the potential of EV-based strategies for fine-tuning CDK5 signaling in AD.

Advances in EV engineering have also enabled the development of multifunctional delivery systems with enhanced targeting and controlled release. For example, EVs engineered to display the phosphotyrosine-binding 2 domain of Fe65, which binds the C-terminal domain of APP, show preferential association with APP-overexpressing neurons in AD models. Loading these EVs with corynoxine-B enhances autophagy and improves cognitive function in APP-overexpressing mice [158]. In another approach, EVs derived from peripherally activated neutrophils are modified using click chemistry to incorporate superparamagnetic iron oxide nanoparticles, encapsulate curcumin, and include a mitochondrial-targeting peptide, together with a matrix metalloproteinase-2-responsive element for controlled cargo release. This multifunctional EV system reduces Aβ production, promotes Aβ clearance, and preserves mitochondrial function, thereby slowing AD progression in vivo [159].

### 6.3. Regulation of EV Biogenesis

EV-mediated transport of pathological proteins is a major driver of AD progression; therefore, targeting the biogenesis and release of EVs, particularly endosome-derived EVs, represents a rational therapeutic strategy. nSMase2 catalyzes the conversion of sphingomyelin to phosphocholine and ceramide and plays a pivotal role in ceramide-dependent EV biogenesis and release. Genetic deletion of nSMase2 in 5XFAD mice reduces brain EV and ceramide levels, attenuates glial activation, decreases Aβ42 plaque burden and tau phosphorylation, and improves learning and memory [163]. Both the classical nSMase2 inhibitor GW4869 and newly developed nSMase2-targeting compounds suppress EV biogenesis and secretion, thereby alleviating cognitive deficits in AD model mice [95,164].

Microglia contribute to AD pathology through EV-mediated tau export. Blockade of the P2X purinergic receptor 7 (P2RX7) inhibits the release of tau-containing microglial EVs. Oral administration of the P2RX7-selective inhibitor GSK1482160 significantly reduces hippocampal accumulation of misfolded tau and EV release in P301S tau mice, leading to improved cognitive performance [165].

Given the diverse and cell-type-specific functions of EVs in AD, effective modulation of EV biogenesis will require strategies that achieve selective targeting of pathogenic EV pathways while preserving homeostatic intercellular communication.

### 6.4. EV-Based and EV-Inspired Material Engineering

EV material engineering is an emerging interdisciplinary field that integrates EV biology with nanotechnology and biomaterials science to modify EV function for biomedical applications, including drug delivery and neurodegenerative disease therapy. hUCB-MSC-EVs generated under three-dimensional graphene scaffold culture conditions display distinct miRNA and protein profiles compared with those produced under conventional two-dimensional conditions [160]. These 3D-conditioned EVs reduce Aβ production in APP-transfected SH-SY5Y cells and in APP/PS1 mice by increasing α-secretase and decreasing β-secretase activity, leading to improved cognitive performance.

In addition to exosome-based systems, other classes of EVs, including microvesicles and engineered membrane-derived vesicles, are increasingly being explored as therapeutic delivery platforms. These vesicles have some unique properties in common with exosomes, such as nano-scale size and biocompatibility; however, they are unique in terms of production scalability and manipulability. Among other types of EVs, cytochalasin B-induced membrane vesicles that are produced by the process of cytoskeletal disruption seem to be an attractive candidate for drug delivery. Recent data revealed that mesenchymal stem cell-derived microvesicles could pass into the brain via the nasal route and accumulate in vulnerable zones of the brain, like the hippocampus and cortex, in the context of AD [166]. These results indicate the therapeutic potential of non-exosomal vesicles as an alternative nanocarrier system for targeted drug delivery in neurodegenerative disorders. Apart from native EVs, another category of nanovesicle that was developed from hybridizing EVs is EV-based nanovesicles, which help improve the loading of cargoes and targeted delivery to desired sites. A biomimetic nanovesicle system was developed using EV membranes isolated from MSCs and liposomes for delivering BACE1 siRNA and TREM2 expression plasmids. For this system, the liposome part has been modified with angipoint-2 peptides to interact with lipoprotein-related protein-1 receptors, allowing more efficient entry to brain tissue and lowering inflammation and Aβ deposition in AD [161]. The above hybrid platforms incorporate the biological characteristics of EVs in combination with the flexible nature of artificial nanocarriers.

The therapeutic application of EVs in the treatment of neurodegenerative diseases was initially shown by using genetically engineered exosomes to deliver siRNA through the blood–brain barrier to silence genes in the brain. In this study, the systemic delivery of RVG-conjugated exosomes carrying BACE1 siRNA successfully suppressed gene expression in neurons. This early study validated the use of EVs as a promising strategy for delivering nucleic acids into the brain and served as a stepping stone for developing new approaches to EV-based therapy. Following this early success, there have been additional advancements in EV engineering to develop stimulus-responsive EVs. Using the biological activity of EVs in conjunction with the photo-cleavable protein mMaple3, the MAPLEX platform permits the cleavage of a linker under blue light exposure, thus facilitating controlled release of cargo within engineered EVs. The MAPLEX approach can deliver transcription factors, Cre recombinase, and dCas9-DNMT3A to silence the BACE1 promoter by means of epigenetic modification to decrease Aβ synthesis [162]. These methods showcase the possibility of designing EV-based systems for gene regulation in a spatiotemporally controlled manner.

While these developments are commendable, there are still many hurdles to overcome when it comes to delivering EV-based drugs to the brain owing to the presence of the blood–brain barrier and the rapid elimination of the therapeutic agents from the body. While surface modifications, novel delivery strategies, and EV-inspired hybrids have enhanced delivery efficiency to the brain, many obstacles still exist in terms of scalability, reproducibility, and safety. Further refinement and validation in animal models will be necessary to transition EV-based bio-material engineering into clinically actionable treatments for AD.

### 6.5. Comparison of Biodistribution and Pharmacokinetics with Conventional Delivery Vectors

Despite encouraging preclinical advances in the use of engineered EVs as nanodelivery systems for AD treatment, there is an urgent need to comparatively assess the biodistribution, brain delivery, and pharmacokinetics of engineered EVs as compared with other established delivery vehicles such as AAVs, liposomes, and LNPs. Preclinical data indicate that EVs could cross the BBB under specific circumstances, partly mediated by receptor-mediated endocytosis and EVs carrying cell-derived surface ligands, and at times possess longer plasma half-lives than typical liposomes [167,168]. In comparison with AAVs, which offer long-term gene transduction in neurons but carry the risk of potential immunogenicity and genomic integration, EVs allow multiple dosing without genetic manipulation [169,170,171]. Liposomes and LNPs deliver nucleic acids and drug molecules effectively, but they usually require chemical or peptide modifications to ensure brain delivery and can also provoke systemic immune responses [172,173]. In addition, engineered EVs with increased targeting capabilities and those derived from neural or immune cells demonstrate greater accumulation in the CNS than unengineered EVs, though peripheral sequestration remains a significant issue [174,175]. It should be noted that direct comparative evaluations between these various systems are scarce, highlighting the importance of establishing quantitative approaches to characterize each system’s unique attributes.

While many studies highlight the therapeutic benefits of miRNA-enriched, drug-loaded, or surface-engineered EVs in AD models, growing evidence indicates that repeated EV administration can induce immune clearance, alter pharmacokinetics, and reduce efficacy. Driedonks et al. demonstrated that accelerated clearance is more strongly associated with repeated dosing than with single high-dose exposure in EV biodistribution studies [176], suggesting an adaptive immune component. Consistently, PEGylated EVs induce anti-PEG IgM after a single intravenous injection in mice, which may accelerate clearance or compromise subsequent dosing [177]. A recent review further emphasized that repeated administration of non-autologous or surface-modified EVs can activate both innate and adaptive immune responses, progressively altering EV pharmacokinetics and biodistribution [178].

With respect to systemic safety, most preclinical studies report minimal overt toxicity, including stable body weight and absence of gross histopathological abnormalities. However, long-term, high-frequency dosing regimens remain poorly characterized. Although chimeric antigen receptor–EV platforms show favorable short-term safety at relatively high doses, data on immune memory, off-target accumulation, and functional consequences in non-CNS tissues remain limited [179], raising the possibility that current preclinical models underappreciate cumulative or delayed toxicities.

Retrograde biodistribution remains an important challenge for EV-mediated CNS delivery. After systemic administration, EVs are rapidly cleared by the reticulo-endothelial system in the liver, spleen, lung, and kidneys, with just a small number of particles accumulating in the brain [180]. Quantitative imaging data also demonstrate that EV biodistribution occurs mainly in the form where roughly 73% of ^89Zr-labeled EVs distribute in the liver 2 h post intra-venous administration while less than 0.1% accumulates in the brain [181]; at the same time, EVs labeled with ^111In localize mainly in the liver (~56%) and spleen (~38%) and have little CNS accumulation [182]. So, although EVs hold a lot of potential for disease therapy, there is still a lot of work that needs to be done to address challenges such as immunogenicity, lack of safety data, and high retention by the reticuloendothelial system before translating EVs into AD therapies. Solving such problems through better targeting approaches and thorough pharmacokinetics studies will be vital in moving EV-based therapies forward in AD.

## 7. Clinical Advances and Remaining Barriers

Studies of EVs in AD have grown immensely fast, demonstrating both the involvement of EVs as vectors of pathological protein spreading and the use of EVs for the identification of biomarkers and as drug delivery systems. EVs can facilitate the spread of toxic Aβ and tau proteins that lead to the spread of AD pathology, yet the question arises whether they play the role of initiators or facilitators of the disease; on the other hand, they offer a novel means to understand the mechanisms of this condition.

Despite increasing pre-clinical interest, the number of registered clinical studies studying the EV-based therapeutic approach for the management of AD is very small, with many being in early recruitment stages. There are two clinical studies that have been set up specifically to assess the effects of EVs in the treatment of AD. One of the studies focuses on the effects of intranasal delivery of allogeneic human adipose-derived MSC EVs in the management of mild-moderate AD, where preliminary results show some improvement in cognition and reduction in hippocampal atrophy after 12 weeks of therapy (NCT04388982) [183]. Another trial assessing the intranasal administration of hUCB-MSC-EVs for AD treatment is ongoing, but efficacy has not been reported (NCT06607900). Both studies use stem-cell-derived EVs with intranasal administration. This is a translational approach that aims to optimize the delivery of EVs to the brain while limiting any adverse systemic side effects.

There are other ongoing clinical investigations that concentrate on EV biomarkers. Immune-captured neuron-enriched EV subfractions from blood plasma have been employed in these investigations. For example, one ongoing study applies spectroscopic analysis to saliva and saliva-derived EVs from healthy controls and AD patients to explore non-invasive biochemical biomarkers (NCT06869135). However, these studies remain in early recruitment phases and have not yet produced peer-reviewed outcome data.

Collectively, these efforts indicate that EVs are entering the translational pipeline, but the field remains at an exploratory stage. Currently, no EV-mediated therapy has been shown to exhibit reproducible efficacy in AD. Several issues that hinder the development of such therapies include difficulties in achieving a standardized protocol for isolating and characterizing EVs, a lack of appropriate dosage and administration protocols, difficulties associated with mass production and quality control, and an inadequate regulatory framework. In addition, the inherent biological diversity of EVs and incomplete knowledge about their biological behaviors in vivo such as distribution and immune recognition make it difficult to translate the promising preclinical data on EV-based therapies to the clinic. Overcoming these hurdles will be critical to realizing the full potential of EVs in transforming their mechanistic and preclinical promise into clinically effective treatments for AD.

## 8. Conclusions and Future Directions

In future studies, advancements in EV therapy for AD will rely on the integration of mechanistic knowledge, engineering ingenuity, and translational success. Elucidation of the mechanisms behind EV production, cargo selection, and selective targeting in aging neurons, neuroinflammation, and protein misfolding is crucial for identifying pathologic versus beneficial EV populations. It is important to determine whether EVs function as the driving forces behind disease mechanisms or merely as the carriers that increase the disease mechanisms to understand their clinical relevance. Also, EVs have to be engineered in such a way that they can be utilized as an integral component of the new therapeutic modalities being developed. Translation of EV-based therapies to the clinic will need multicenter randomized trials enabled by uniform production, pharmacokinetic analysis, biodistribution mapping, and toxicity testing. Collectively, these endeavors will determine whether EVs can develop from experimental tools into clinical realities for AD diagnosis and treatment.

## Figures and Tables

**Table 1:** Changes in the characteristics of EVs from AD patients.

EVs Source	Sample Source	Biomarkers	Dynamic Changes	References
Peripheral blood	AD patients	Aβ42	Increased	[30]
		AACT, C4BPα	Increased	[31]
		C1QC, C9, CFAH, KVD30, GP1BB, RSU1, A0A0G2JRQ6	Increased	[32]
		A2MG, ADA10, A1AG2, IGHA1, HV428	Decreased	
		miR-106a-5p, miR-16-5p, miR-223-3p, miR-25-3p, miR-30b-5p, miR-92a-3p, miR-451a	Increased	[33]
		miR-22-3p, miR-378a-3p	Decreased	[34]
		miR-30b-5p	Increased	
	MCI, DAT, PDD and VaD patients	miR-135a, miR-384	Increased	[35]
		miR-193b	Decreased	
Peripheral blood neuronal	AD and aMCI patients	Aβ42, t-tau, p-tau	Increased	[36,37,38]
	FTD and AD patients	synaptic proteins	Decreased	[39,40,41]
	AD and MCI patients	C7	Increased	[42]
		ZYX	Decreased	
	AD patients	TDP-43	Increased	[43]
		miR-29c-5p, miR-143-3p, miR-335-5p, miR-485-5p	Increased	[44]
		miR-138-5p, miR-342-3p	Decreased	
	SCD patients	miR-29c-3p	Increased	[45]
	AD and aMCI patients	miR-384	Increased	[46]
	AD patients	miR-212-3p, miR-132-3p	Decreased	[47]
Cerebrospinal fluid	AD patients	miR-125b-5p	Increased	[48]
		miR-451a, miR-605-5p	Decreased	
		miR-27a-3p, miR-30a-5p, miR-34c, piR-019949, piR-020364	Increased	[49]
		piR-019324	Decreased	
Urine	5XFAD mouse	Aoah	Increased	[50]
		NEU1, Anxa2, Clusterin, Ly86	Decreased	
		miR-196b-5p, miR-34a-5p, miR-376b-3p	Increased	[51]
		miR-339-3p, miR-677-5p, miR-721	Decreased	
Astrocyte	AD patients	C1q, C4b, C3d, Factor B, Factor D, Fragment Bb, C3b, C5b-C9 TCC	Increased	[52]
		CD59, CD46, CR1, DAF	Decreased	
		BACE-1, sAPPβ	Increased	[53]
		ceramide, PAR-4	Increased	[54]
Microglia		P2RY12, TMEM119, DHA	Decreased	[55,56]
		FTH1, TREM2, tau, free cholesterol	Increased	

aMCI, amnesic mild cognitive impairment; FTD, frontotemporal dementia; C7, complement component 7; ZYX, Zyxin; TDP-43, TAR DNA binding protein 43; SCD, subjective cognitive decline; AACT, Aβ-binding protein α-1-antichymotrypsin; C4BPα, C4b-binding protein alpha chain; C1QC, complement C1q subcomponent subunit C; C9, complement component C9; CFAH, complement factor H; KVD30, immunoglobulin kappa variable 2D-30; GP1BB, platelet glycoprotein Ib beta chain; RSU1, Ras suppressor protein 1; A0A0G2JRQ6; Ig-like domain-containing protein; A2MG, alpha-2-macroglobulin; ADA10, disintegrin and metalloproteinase domain 10; A1AG2, alpha-1-acid glycoprotein 2; IGHA1, immunoglobulin heavy constant alpha 1; HV428, immunoglobulin heavy variable 4-28; DAT, dementia of Alzheimer-type; PDD, Parkinson’s disease with dementia; VaD, vascular dementia; Aoah, acyloxyacyl hydrolase; NEU1, neuraminidase 1; Anxa2, Annexin 2; Ly86, lymphocyte antigen 86; C1q, complement component 1q; C4b, complement component 4b; C3d, complement component 3d; Fragment Bb, complement fragment Bb; C3b, complement component 3b; C5b-C9 TCC, C5b-C9 terminal complement complex; CR1, complement receptor type 1; DAF, decay-accelerating factor; BACE-1, beta-site amyloid-precursor-protein-cleaving enzyme 1; sAPPβ, soluble amyloid precursor protein β; PAR-4, prostate apoptosis response 4; P2RY12, purinergic 2Y receptor 12; TMEM119, transmembrane protein 119; DHA, docosahexaenoic acid; FTH1, ferritin heavy chain-1; TREM2, triggering receptor expressed on myeloid cells 2. Biomarkers are listed across different stages of validation, including exploratory, validation-stage, and advanced candidates.

**Table 2:** Differentially expressed EV miRNAs and AD-associated target genes in AD patients.

Expression Change	miRNA	DisGeNET to AD
Increased	miR-106a-5p	PRNP, TFAM, DPYSL2, SOD2
	miR-16-5p	IGF2R, TPI1, ATP5A1, F2, CLU, BACE1, GSK3B, PRNP, BCL2, IGF1R, HMOX1
	miR-223-3p	IGF1R, SLC2A4
	miR-25-3p	
	miR-30b-5p	EIF2S1, CASP3, BCL2
	miR-92a-3p	ATP5A1, GSK3B, ENO1, TFAM, IGF1R
	miR-451a	BCL2
	miR-135a	BCL2, ARC
	miR-384	
	miR-29c-5p	
	miR-143-3p	TNF, IGF1R, BCL2
	miR-335-5p	GATA1, ABCA7, CD33, GAPDHS, NOS3, EPHA1, HMOX1, IGF1R
	miR-485-5p	F2, CLU, IGF1
	miR-29c-3p	BACE1, BCL2
	miR-384	
	miR-125b-5p	BCL2, BAX, TNF, IGF1R
	miR-27a-3p	GSK3B, IGF1
	miR-34c-5p	MAPT, GSK3B, BCL2
	miR-196b-5p	CALM1, BCL2
	miR-34a-5p	TREM2, CASP3, BCL2, TFAM, BAX, IGF1R, TNF
	miR-376b-3p	
Decreased	miR-22-3p	ENO1, VSNL1
	miR-378a-3p	ENO1, IGF1R
	miR-193b	
	miR-138-5p	CASP3, IGF1R
	miR-342-3p	SOD2, IGF1R, IGF2R
	miR-212-3p	SOD2, ACHE
	miR-132-3p	GSK3B
	miR-605-5p	
	miR-339-3p	
	miR-677-5p	
	miR-721	

PRNP: prion protein; TFAM: transcription factor A, mitochondrial; ATP5A1: ATP synthase F1 subunit alpha; BACE1: Beta-secretase 1; BCL2: BCL2 apoptosis regulator; CLU: clusterin; F2: coagulation factor II, thrombin; GSK3B: glycogen synthase kinase 3 beta; HMOX1: heme oxygenase 1; IGF1R: insulin-like growth factor 1 receptor; IGF2R: insulin-like growth factor 2 receptor; TPI1: triosephosphate isomerase 1; IGF1: insulin-like growth factor 1; ENO1: enolase 1; DPYSL2: dihydropyrimidinase like 2; EIF2S1: eukaryotic translation initiation factor 2 subunit alpha; SOD2: superoxide dismutase 2; BAX: BCL2 associated X, apoptosis regulator; CASP3: caspase 3; TNF: tumor necrosis factor; TREM2: triggering receptor expressed on myeloid cells 2; SLC2A4: solute carrier family 2 member 4 (GLUT4); ABCA7: ATP binding cassette subfamily A member 7; EPHA1: EPH receptor A1; GAPDHS: glyceraldehyde-3-phosphate dehydrogenase, spermatogenic; GATA1: GATA binding protein 1; NOS3: nitric oxide synthase 3; CALM1: calmodulin 1; ARC: activity regulated cytoskeleton associated protein; MAPT: microtubule associated protein tau; VSNL1: visinin-like 1; ACHE: acetylcholinesterase.

**Table 3:** Summary of EV bioengineering strategies for therapeutic applications in AD.

EVs Source	Bioengineering Methods	Bioengineering Content	Functional Effects	References
DC	cell virus transfection	CD47	immune escape	[149]
		CCK/SST	brain targeting	
		miR-29b-2	suppress PSEN1	
BMSCs		miR-29a/miR-29b	suppress BACE1	[150]
AMSCs		miR-22	suppress neuroinflammation	[151]
BMSCs	surface modification	RVG peptides	enhanced CNS targeting	[152]
AMSCs	endogenous loading	CoQ10	upregulation of BDNF and SOX2	[153]
BMSCs	electroporation	olesoxime/resveratrol	suppress oxidative stress and apoptosis	[154]
microglia	ultrasonication loading	berberine/palmatine	suppress neuroinflammation	[155]
astrocyte	cell-drug co-incubation	catalpol/tetramethylpyrazine	promote CDK5-mediated STAT3 phosphorylation	[156]
plasma	co-incubation and ultrasonication	quercetin	suppress CDK5-mediated tau phosphorylation and NFTs formation	[157]
NDEs	plasmid transfection	Fe65	targeting APP-overexpressing neurons	[158]
	ultrasonication loading	corynoxine-B	enhance neuronal autophagy	
activated neutrophil	chemical coupling	N3-GPLGLAGC-HS	MMP2 response element-controlled drug release	[159]
	chemical coupling	SPION	targeting Aβ deposition	
	membrane insertion	DSPE-PEG2000-MitP	enhance mitochondrial targeting	
	hydrophobic diffusion and click chemistry	curcumin	reduce Aβ production	
hUCB-MSCs	3D graphene scaffold culture		upregulation of α-secretase and downregulation of β-secretase	[160]
MSCs	lipid insertion	angiopep-2 peptides	targeting the LRP1 receptor to cross the BBB	[161]
	aqueous core encapsulation and electrostatic interaction	BACE1 siRNA/TREM2 plasmid	reduce Aβ production and suppress neuroinflammation	
	extrusion	lipid nanovesicles	delivery of target genes	
HEK-293T cells	plasmid transfection	mMaple3-CD9	photosensitive release and exosome membrane anchoring	[162]
	mMaple3-CD9-mediated loading	dCas9-D3A	BACE1 DNA methylation	

DC, dendritic cells; CCK, cholecystokinin; SST, somatostatin; PSEN1, presenilin 1; RVG, rabies virus glycoprotein; CoQ10, coenzyme Q10; BDNF, brain-derived neurotrophic factor; SOX2, SRY-box transcription factor 2; CDK5, cyclin-dependent kinase 5; STAT3, signal transducer and activator of transcription 3; MMP2, matrix metallopeptidase 2; SPION, superparamagnetic iron oxide nanoparticles; LRP1, lipoprotein receptor-related protein 1; BACE1, β-site amyloid precursor protein cleaving enzyme 1; TREM2, triggering receptor expressed on myeloid cells-2; BBB, blood–brain barrier.

## Data Availability

No new data were created or analyzed in this study.
